# DHA but Not EPA Emulsions Preserve Neurological and Mitochondrial Function after Brain Hypoxia-Ischemia in Neonatal Mice

**DOI:** 10.1371/journal.pone.0160870

**Published:** 2016-08-11

**Authors:** Korapat Mayurasakorn, Zoya V. Niatsetskaya, Sergey A. Sosunov, Jill J. Williams, Hylde Zirpoli, Iliyan Vlasakov, Richard J. Deckelbaum, Vadim S. Ten

**Affiliations:** 1 Institute of Human Nutrition, Columbia University, New York, New York, United States of America; 2 Department of Pediatrics, Columbia University, New York, New York, United States of America; 3 Center for Experimental Therapeutics and Reperfusion Injury, Department of Anesthesiology, Perioperative and Pain Medicine, Harvard Institutes of Medicine, Brigham and Women’s Hospital and Harvard Medical School, Boston, MA, United States of America; Fraunhofer Research Institution of Marine Biotechnology, GERMANY

## Abstract

**Background and Purpose:**

Treatment with triglyceride emulsions of docosahexaenoic acid (tri-DHA) protected neonatal mice against hypoxia-ischemia (HI) brain injury. The mechanism of this neuroprotection remains unclear. We hypothesized that administration of tri-DHA enriches HI-brains with DHA/DHA metabolites. This reduces Ca^2+^-induced mitochondrial membrane permeabilization and attenuates brain injury.

**Methods:**

10-day-old C57BL/6J mice following HI-brain injury received tri-DHA, tri-EPA or vehicle. At 4–5 hours of reperfusion, mitochondrial fatty acid composition and Ca^2+^ buffering capacity were analyzed. At 24 hours and at 8–9 weeks of recovery, oxidative injury, neurofunctional and neuropathological outcomes were evaluated. *In vitro*, hyperoxia-induced mitochondrial generation of reactive oxygen species (ROS) and Ca^2+^ buffering capacity were measured in the presence or absence of DHA or EPA.

**Results:**

Only post-treatment with tri-DHA reduced oxidative damage and improved short- and long-term neurological outcomes. This was associated with increased content of DHA in brain mitochondria and DHA-derived bioactive metabolites in cerebral tissue. After tri-DHA administration HI mitochondria were resistant to Ca^2+^-induced membrane permeabilization. *In vitro*, hyperoxia increased mitochondrial ROS production and reduced Ca^2+^ buffering capacity; DHA, but not EPA, significantly attenuated these effects of hyperoxia.

**Conclusions:**

Post-treatment with tri-DHA resulted in significant accumulation of DHA and DHA derived bioactive metabolites in the HI-brain. This was associated with improved mitochondrial tolerance to Ca^2+^-induced permeabilization, reduced oxidative brain injury and permanent neuroprotection. Interaction of DHA with mitochondria alters ROS release and improves Ca^2+^ buffering capacity. This may account for neuroprotective action of post-HI administration of tri-DHA.

## Introduction

Neonatal hypoxia-ischemia (HI) brain injury significantly contributes to neurological mortality in children [1]. With a prevalence of 1 per 3500 live births, more than 25% of asphyxiated infants develop life-long neurological impairments.^1^ Various mechanisms of neuronal damage following HI have been proposed.[2–4]. Within minutes of acute oxygen and nutrient deprivation, early energy failure initiates cellular dysfunction, which without reoxygenation/reperfusion, eventuates in cellular death. Upon reperfusion, the bioenergetics of the post-ischemic tissue almost fully recovers. However, over the next few hours, secondary energy failure takes place and drives the evolution of necrosis and apoptosis [5,6] Mitochondrial dysfunction is known mechanism of secondary energy failure [2,6] During reperfusion, mitochondria are overloaded with Ca^2+^. This triggers the opening of permeability transition pores (mPTP) in the mitochondrial inner membrane which arrests ATP production secondary to loss of the proton motive force and inhibition of the respiratory chain [2] At the same time, BAX and other pro-apoptotic proteins permeabilize the outer mitochondrial membrane, causing the release of apoptotic proteins [7]

N-3 polyunsaturated fatty acids (n-3 FAs) affect membrane composition and properties and have the potential to enhance endogenous protective mechanisms attenuating brain injury [3]. In mouse models of stroke, docosahexaenoic acid (DHA) and neuroprotectin D1 (NPD1), a bioactive derivative of DHA, significantly decreased brain tissue loss [8,9]. This neuroprotection was attributed to anti-inflammatory actions of NPD1 and DHA. DHA concentrates in the membranes of brain grey matter and increases membrane fluidity [10]. After ischemic stroke, DHA is liberated from membrane phospholipids into the cytoplasm by phospholipase A2 and can be converted to NPD1 [11].

We recently reported that administration of n-3 TG enriched with both DHA and eicosapentaenoic acid (EPA), immediately prior to and after HI, decreased brain injury in neonatal mice [12] Furthermore, when we used only DHA- or only EPA-enriched TG emulsions (tri-DHA or tri-EPA, respectively), only tri-DHA exerted neuroprotection[12]. It has also been reported that DHA, but not EPA, increased the tolerance of myocardial mitochondria to Ca^2+^-induced mPTP opening [13] We hypothesized that tri-DHA but not tri-EPA exerts long-term or permanent neuroprotection since DHA, but not EPA, interacts with cerebral mitochondria. We also hypothesized that DHA inhibits post-ischemic mPTP opening and therefore attenuates secondary mitochondrial dysfunction. To test these hypotheses we compared the effects of tri-DHA versus tri-EPA on brain mitochondria in the HI-reperfusion paradigm. By evaluation of short- and long-term recovery, we also determined whether, in neonatal mice, acute post-treatment with tri-DHA exerts permanent neuroprotection.

## Materials & Methods

### Lipid emulsions

We prepared VLDL-sized emulsions with TG oil containing either only DHA or only EPA and egg yolk phospholipid, as previously detailed [12]. The emulsions were analyzed for the amount of TG and phospholipids (Wako Chemicals, Inc.). The TG: phospholipid mass ratio was 5.0 ± 1.0, similar to that of VLDL-sized particles.

### Unilateral cerebral hypoxia-ischemia injury

All studies were conducted according to protocols approved by the Columbia University Institutional Animal Care and Use Committee (IACUC) and in accordance with the AAALAC guidelines. Three-day-old C57BL/6J neonatal mice with their dams were purchased from Jackson Laboratories (Bar Harbor). At 10 days of age (p10), mice were subjected to HI-insult as previously described [12,14] Briefly, HI brain injury was induced by permanent ligation of the right common carotid artery under 2% isoflurane anesthesia. After 1.5h of recovery, mice were exposed to hypoxic (humidified 8% O_2_/ 92% N_2_, Tech Air Inc., NY) insult for 15 min, at 37± 0.3°C [12,14]

### Treatment protocol

The doses (0.375 g n-3 TG/kg/dose) and the route of administration (intraperitoneum, IP) were selected based on our previous work [12] The first dose was administered immediately after HI and the second dose 1h after the first dose. Normal saline was used as vehicle (Veh). Three groups were compared: HI+Veh; HI+tri-EPA; and HI+tri-DHA.

### Neurofunctional outcomes

All neurofunctional outcomes were compared between study groups and naïve littermates. Short-term neurofunctional outcomes were assessed at 24h after HI by evaluation of righting reflex and negative geotaxis reflex performance, as previously detailed [14]. Not all mice were assessed for neonatal reflex performance, because in one set of experiments neurofunctional evaluation was missed.

Long-term neurofunctional outcome were defined by navigational memory performance in the Morris water-maze test in adult mice (8 weeks after neonatal HI), as previously described [15] Briefly, the landing platform (7.5 cm in diameter) was 0.5 cm submerged in one of four quadrants in the pool with diameter 150 cm. For 3 consecutive days, animals were given three attempts daily to learn to find the platform within 120 sec. The total latency time recorded over three training days was used to assess navigational learning. On the day of navigational memory testing, the landing platform was removed and the time spent in the “landing” quadrant was recorded (allotted time: 60 sec).

### Neuropathological outcomes

At 24h of reperfusion, mice were sacrificed, brains were coronally sectioned and stained with tri-phenyl-tetrazolium chloride (TTC). Infarcted area was traced in digital images using NIH-image 4.1 and expressed as % of the ipsilateral hemisphere. Due to accidental loss of specimens from the mice which were tested for navigational memory, long-term neuropathological outcomes were assessed in a separate cohort of adult HI-mice at 8–9 weeks following HI. Brains were coronally sectioned and Nissl-stained as previously described [16] The extent of injury was defined by the % of residual brain tissue volume in the ipsilateral hemisphere in relation to the contralateral hemisphere.

### Assessment of DHA derived bioactive metabolites in brain tissue

To determine whether post-treatment with tri-DHA changes the content of DHA derived bioactive metabolites, at two hours of reperfusion, cerebral concentrations of NPD1 and Resolvins D series (RvD1-6) were measured in HI-mice treated with tri-DHA or vehicle. All samples for LC-MS-MS-based lipidomics were subject to solid-phase extraction as described [17] Prior to sample extraction, d8-5-HETE, d5-RvD2, d5-LXA_4_, d4-LTB_4_, d4-PGE_2_ internal standards (500 pg each) were added to facilitate quantification. Extracted samples were analyzed by a liquid chromatography-ultraviolet-tandem mass spectrometry system, QTrap 5500 (AB Sciex) equipped with a Shimadzu LC-20AD HPLC (Tokyo, Japan). A Poroshell 120 EC-18 column (100 mm × 4.6 mm × 2.7 μm; Agilent Technologies, Santa Clara, CA, USA) was kept in a column oven maintained at 50°C, and lipid mediators (LMs) were eluted with a gradient of methanol/water/acetic acid from 55:45:0.01 (v/v/v) to 100:0:0.01 at 0.5 mL/min flow rate. To monitor and quantify the levels of targeted LMs, multiple reaction monitoring (MRM) was used with MS/MS matching signature ion fragments for each molecule (six diagnostic ions and calibration curves). Concentrations of DHA metabolites were expressed in pg/300 mg of brain tissue.

### In vitro studies

In vitro experiments determined whether DHA or EPA directly interacts with brain mitochondria and alters mitochondrial response to oxidative stress. To mimic post-ischemic oxidative stress associated with reintroduction of oxygen (reperfusion), mitochondria isolated from p10 mice were incubated in hyperoxic (paO_2_ ≈ 470 mm Hg) buffer as described [6]. The rate of mitochondrial reactive oxygen species (ROS) emission and mitochondrial Ca^2+^ buffering capacity was assessed in the presence or absence of complexes; DHA: albumin (7:1 molar ratio) or EPA:albumin (7:1 molar ratio) or DHA:albumin (molar ratio 1:1). In brief, 150 μM of DHA or EPA in ethanol was mixed in 1% BSA or 0.147% BSA, which provided a FFA:albumin molar ratio of 1:1 or 7:1, respectively. 100 μg of isolated mitochondria in 100 μl buffer was incubated with 2 μl 0.15 mM DHA or EPA at different FFA:albumin molar ratios for 30 min in ice followed by re-suspension in 1 ml buffer containing 0.2% BSA. For mitochondrial FA content measurement samples were spun down (10,000 g, 10 min). For mitochondrial functional studies (ROS generation rate and Ca^2+^ buffering capacity) samples were free of re-suspension buffer. DHA or EPA displacement from albumin and interaction with phospholipids differs depending on the albumin:FFA molar ratio;^17^ therefore, albumin oversaturated with DHA or EPA (7:1 ratio) would readily release FFA. In contrast, release of FFA from undersaturated albumin (1:1 ratio) would be limited.

### Assessments of mitochondrial function

#### Mitochondrial preparation

Mitochondrial functions were studied using *ex-vivo* isolated brain mitochondria in HI+Veh, HI+tri-EPA or HI+tri-DHA mice and naïve littermates. At 4–5h of reperfusion (the time of secondary energy failure in this model),^6^ mice were euthanized by decapitation and the ipsilateral hemispheres were homogenized in buffer (225 mM mannitol, 75 mM sucrose, 1 mM EGTA, 5mM HEPES (pH 7.2), 1 mg/ml BSA). Mitochondria were isolated as previously described [6].

#### Mitochondrial fatty acyl (FA) composition

At 4-5h of reperfusion, *ex-vivo* isolated brain mitochondria and mitochondria *in-vitro* exposed to DHA:albumin or EPA:albumin complexes were examined for their FA composition using gas chromatography (GC) according to the method of Lepage and Roy [18]. Briefly, 1.5 ml of methanol-acetyl chloride (20:1, vol/vol) solution, 1.5 μl of butylated hydroxyl toluene (20 mM) and 95 μL of hexane were added to 75 μg of mitochondrial proteins. The clear *n*-hexane top layer containing FA methyl esters (FAMEs) was analyzed using a Hewlett-Packard 5890 Series II gas chromatograph. DHA content was expressed as a percentage of total FA, identified by comparison with FA standards.

Mitochondrial ROS production was defined as the rate of H_2_O_2_ emission measured, as described^6^, in isolated mitochondria. Briefly, 50 μg mitochondrial protein was incubated in 1 ml 10mM Tris-MOPS buffer, containing 5 mM succinate, 10 μM Amplex Ultrared (Invitrogen), and 4 U/ml horse radish peroxidase (HRP, Sigma). Changes in H_2_O_2_ fluorescence were recorded using Hitachi 7000 spectrofluorimeter (555 nm excitation and 581 nm emission). The rates of H_2_O_2_ emission were expressed in pmol H_2_O_2_/mg mitochondrial protein /min.

Mitochondrial calcium buffering capacity was measured as described [6]. Briefly, mitochondria (50 μg protein/ml) in 10 mM Tris-MOPS buffer were supplemented with 10 μM 5N-calcium green, 5mM succinate and 2.5 mM glutamate. Mitochondria were repeatedly supplemented with 10 nmol CaCl_2_ every 50 sec until spontaneously released Ca^2+^ indicated an opening of the mPTP. The amount of calcium required for mPTP opening was expressed in nmoles/mg mitochondrial protein.

### Immunohistochemical assessment of oxidative brain injury

In experimental mice, brains were removed and coronal sections (20 μm) were incubated with rabbit monoclonal antibody to 3-nitrotyrosine and to microtubule-associated protein 2 (MAP2) and Nissl for counterstain. Using Image Pro-Plus 4.5 (Media Cybernetics) the 3-nitrotyrosine immunoreactivity was analyzed by the count of immunopositive cells in 5 nonadjacent fields (40X) of injured cortex at 3 different bregma levels. The mean ratio of immunopositive cell / total number of cells per mouse was used for statistical analysis.

## Statistical Analyses

Data are mean ± SEM. One-Way ANOVA followed by Fisher’s post-hoc analysis was used to compare the differences in neurofunctional outcomes and mitochondrial functions among naïve and experimental groups. Statistical significance was determined at *p* ≤ 0.05.

## Results

### Tri-DHA but not with tri-EPA exerts short- and long-term neuroprotection

**Fig 1:**
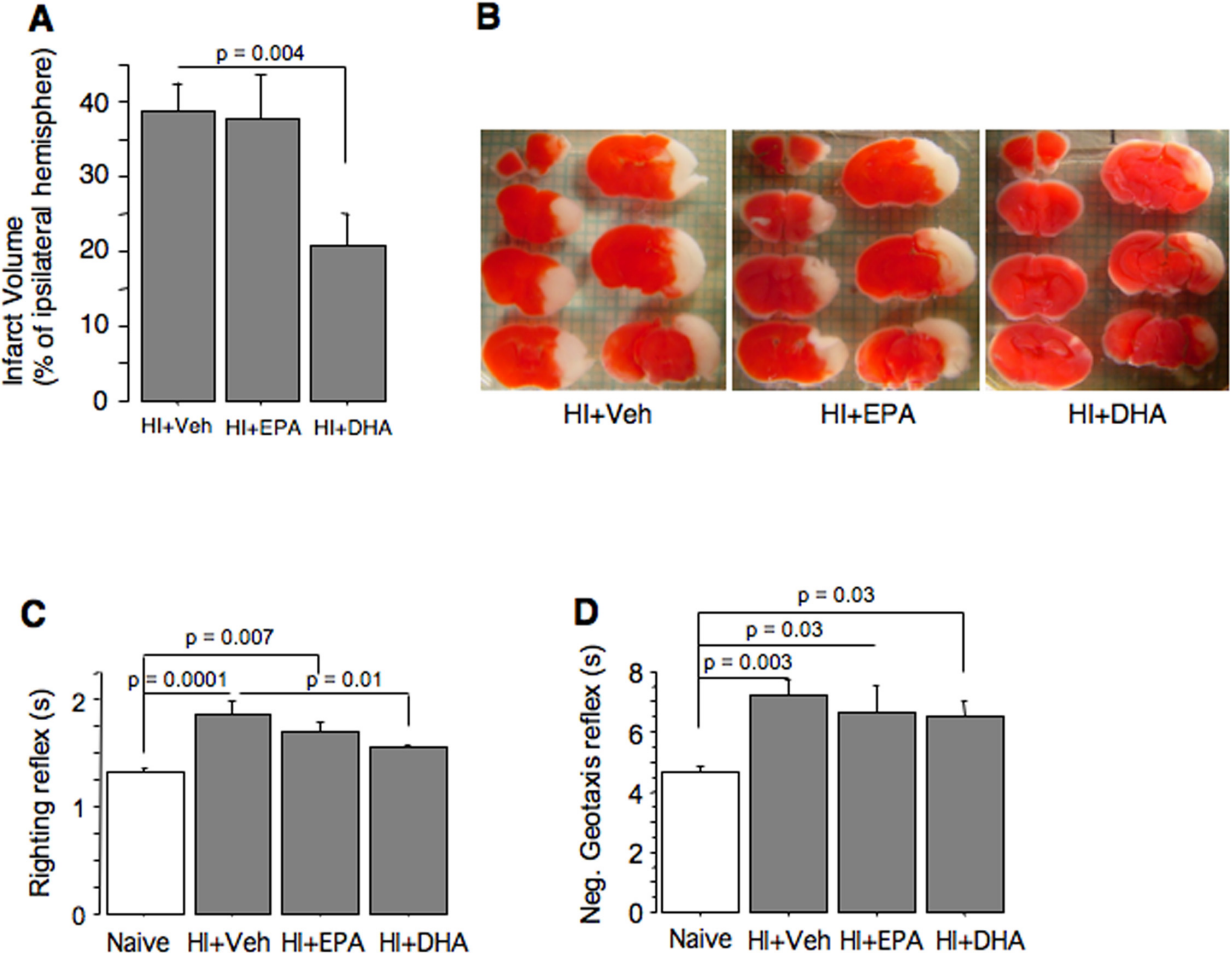
Short term neurological outcomes. (A) Infarct volumes in HI+Veh (n = 30), HI+tri-EPA (n = 20), or HI+tri-DHA (n = 20) mice. (B) Representative TTC-stained cerebral sections from the same groups of mice. (C) Righting, and (D) Negative geotaxis reflex performance in naïve (n = 12); Veh+HI (n = 18); HI+tri-EPA (n = 13); and HI+tri-DHA (n = 18) mice.

At 24h of reperfusion only HI+tri-DHA mice exhibited a significant reduction in their cerebral infarct volumes (Fig 1A and 1B). **Fig** Sensorimotor reflex performance was significantly poorer in HI+Veh mice compared to naïves; both righting and negative geotaxis reflex performance was sluggish (Fig 1C and 1D). Treatment with tri-DHA significantly improved righting reflex performance compared to the HI+Veh littermates (Fig 1C). However, neither HI+tri-DHA nor HI+tri-EPA resulted in significant improvement of negative geotaxis reflex performance (Fig 1D).

**Fig 2:**
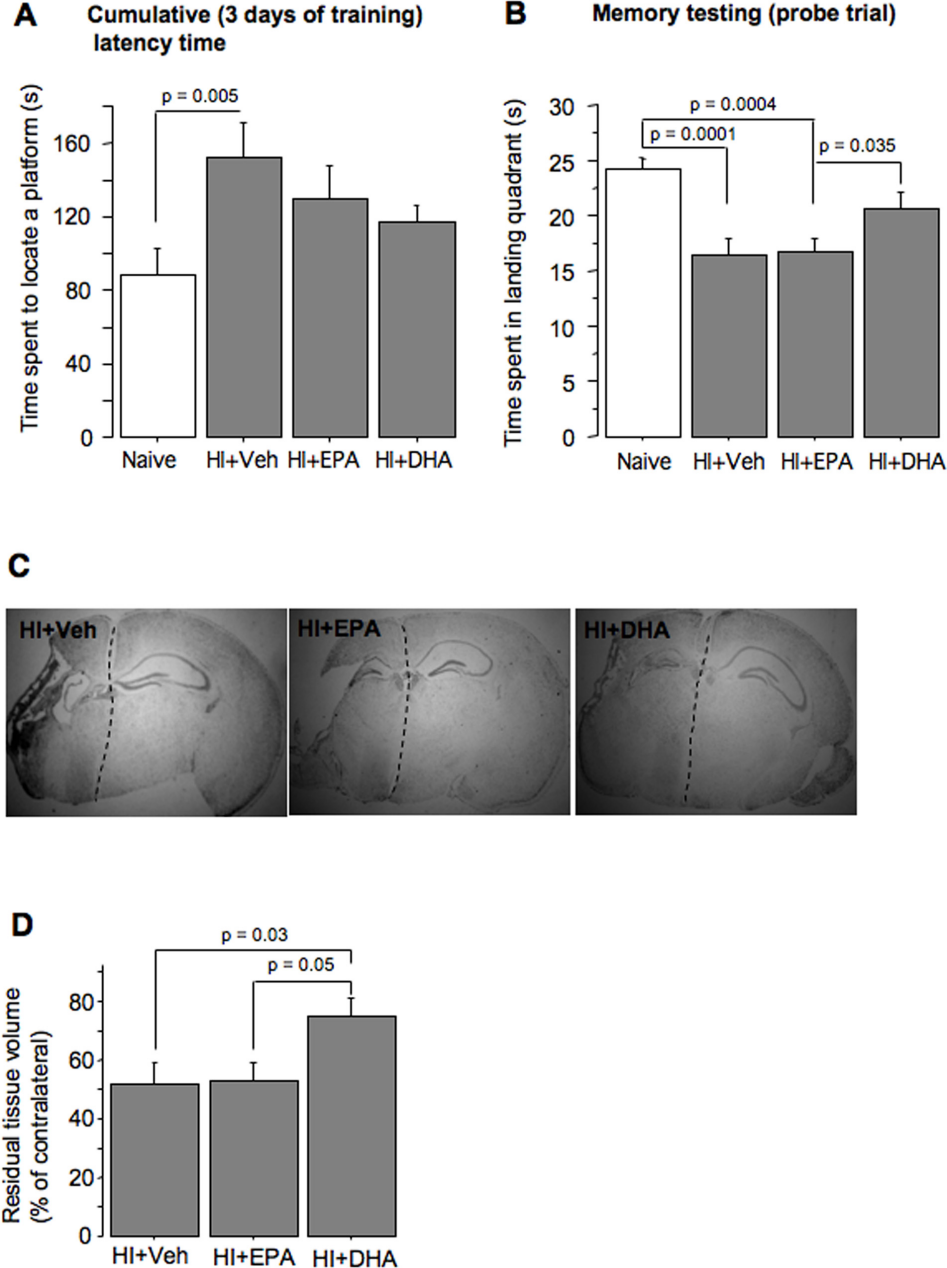
Long term neurological outcomes. (A) Cumulative latency time over 3 days of training and (B) Navigational memory performance in Naive (n = 16); HI+Veh (n = 20); HI+tri-EPA (n = 16) and HI+tri-DHA (n = 22) adult mice. (C) and (D) Nissl-stained cerebral coronal sections and residual ipsilateral hemisphere volume in the adult HI+Veh (n = 6), HI+EPA (n = 5) or HI+tri-DHA (n = 5) mice.

At 8–9 weeks after HI, compared to naïve littermates, adult HI+Veh mice exhibited significant delays in locating a platform during spatial navigation training (Fig 2A). HI+tri-DHA or HI+tri-EPA mice exhibited only a tendency toward improved spatial learning compared to HI+Veh littermates (Fig 2A). However, navigational memory, which was significantly poorer in the HI+Veh mice compared to naïve mice, was significantly improved only in HI+tri-DHA mice (Fig 2B),S1 Data. Neuroanatomical analysis demonstrated a significantly greater preservation of the ipsilateral hemisphere in the HI+tri-DHA mice compared to the HI+tri-EPA or HI+Veh group (Fig 2C and 2D).

### Post-HI administration of tri-DHA increased DHA content and preserved Ca^2+^ buffering capacity in cerebral mitochondria

**Fig 3:**
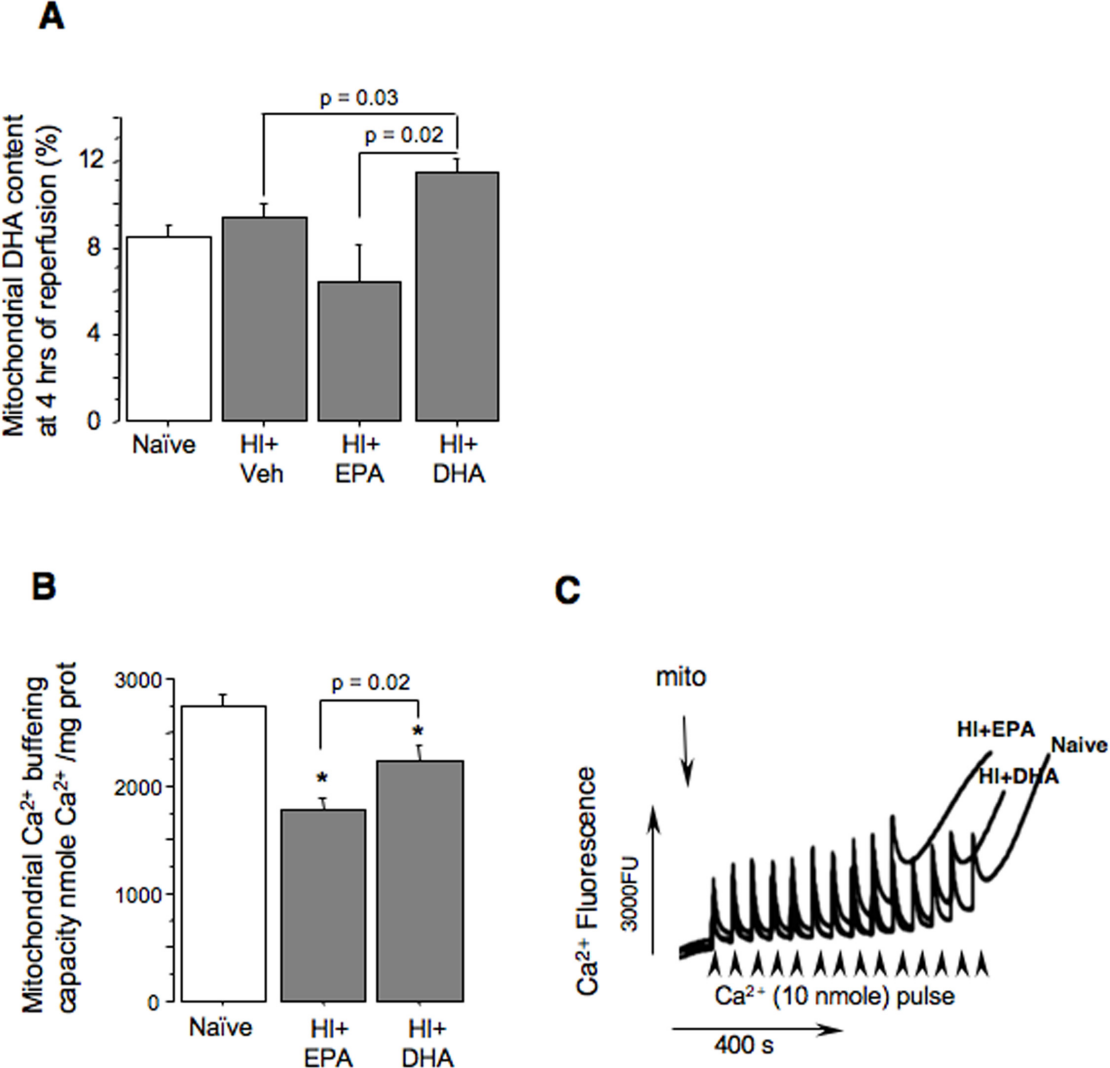
Mitochondrial DHA content and function following HI-insult. (A) Mitochondrial DHA content in Naïve (n = 8); Veh+HI (n = 11); HI+tri-EPA (n = 4); and HI+tri-DHA (n = 12) mice. (B) and (C) Mitochondrial Ca^2+^ buffering capacity in Naïve (n = 7), HI+tri-EPA (n = 6) and HI+tri-DHA (n = 8) mice with representative tracings. * p < 0.05 compared to naives.

Chronic dietary supplementation with n-3 FAs alters mitochondrial phospholipid FA composition which may be beneficial in conditions such as HI [19]. We questioned whether neuroprotection afforded by acute administration of tri-DHA is exerted through modulation of DHA content in brain mitochondria following HI. Compared to naïve mice, the HI+Veh group did not show significant changes in DHA content in brain mitochondria (Fig 3A). As expected, HI+tri-EPA also did not affect mitochondrial DHA content. However, HI+tri-DHA significantly increased mitochondrial DHA content (Fig 3A), the event associated with greater preservation of mitochondrial Ca^2+^ buffering capacity compared to HI+tri-EPA littermates (Fig 3B and 3C). There were no changes in mitochondrial EPA content in the HI+tri-EPA group (data not shown).

### Interaction of brain mitochondria with DHA increased mitochondrial DHA content and improved Ca^2+^ buffering capacity impaired by oxidative stress in vitro

**Fig 4:**
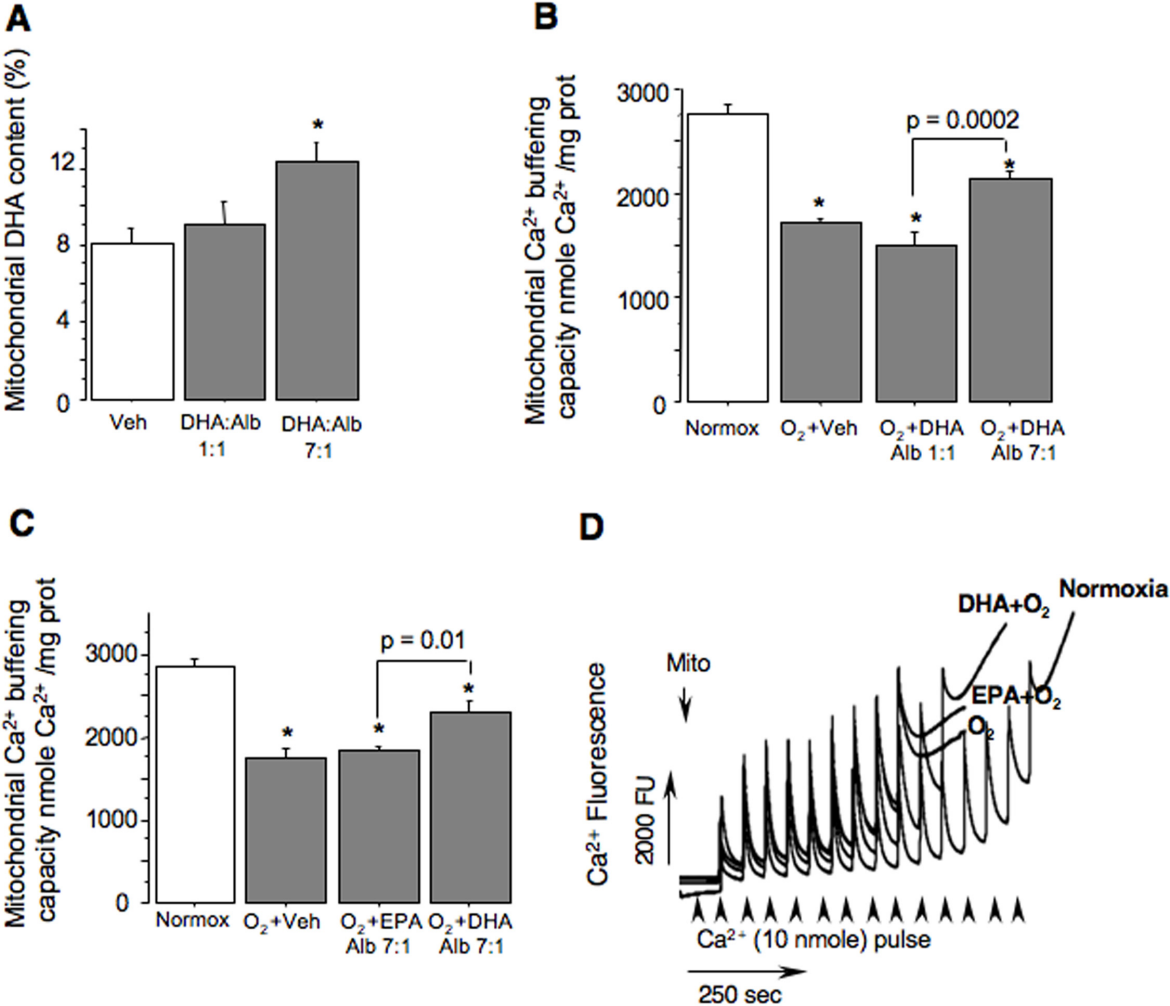
Mitochondrial DHA content and function following hyperoxic stress in vitro. (A) Mitochondrial DHA content following in vitro incubation with Veh, DHA:Alb 1:1, and DHA:Alb 7:1. * p < 0.05. (B) Mitochondrial Ca^2+^ buffering capacity: Normoxia, O_2_+Veh; O_2_ +DHA:Alb 1:1; and O_2_ +DHA:Alb 7:1. * p ≤ 0.0005. (C) Mitochondrial Ca^2+^ buffering capacity in Normoxia; O_2_+Veh; O_2_ +EPA:Alb 7:1; and O_2_ +DHA:Alb 7:1. * p < 0.05. (D) Representative tracings of mitochondrial Ca^2+^ buffering capacity. Groups are indicated. N = 4 in all groups.

To determine if DHA incorporates into mitochondria upon direct interaction, we incubated cerebral mitochondria with a DHA:albumin complexes. At a DHA:albumin molar ratio of 1:1, no mitochondrial DHA enrichment was detected (Fig 4A). However, when the DHA:albumin molar ratio was increased to 7:1, mitochondrial DHA content increased significantly (Fig 4A). Next we tested if mitochondrial DHA enrichment translates to functional benefits in organelles exposed to oxidative stress in vitro. Hyperoxic exposure significantly reduced mitochondrial tolerance to Ca^2+^-induced mPTP opening. Neither the presence of EPA:albumin 7:1, nor the presence of DHA:albumin 1:1, preserved Ca^2+^ buffering capacity after hyperoxia induced damage (Fig 4B–4D). Only exposure to DHA:albumin 7:1 significantly preserved Ca^2+^ buffering capacity, suggesting that, similar to our observations in vivo, increased mitochondrial DHA content is linked to improved mitochondrial Ca^2+^ handling.

**Fig 5:**
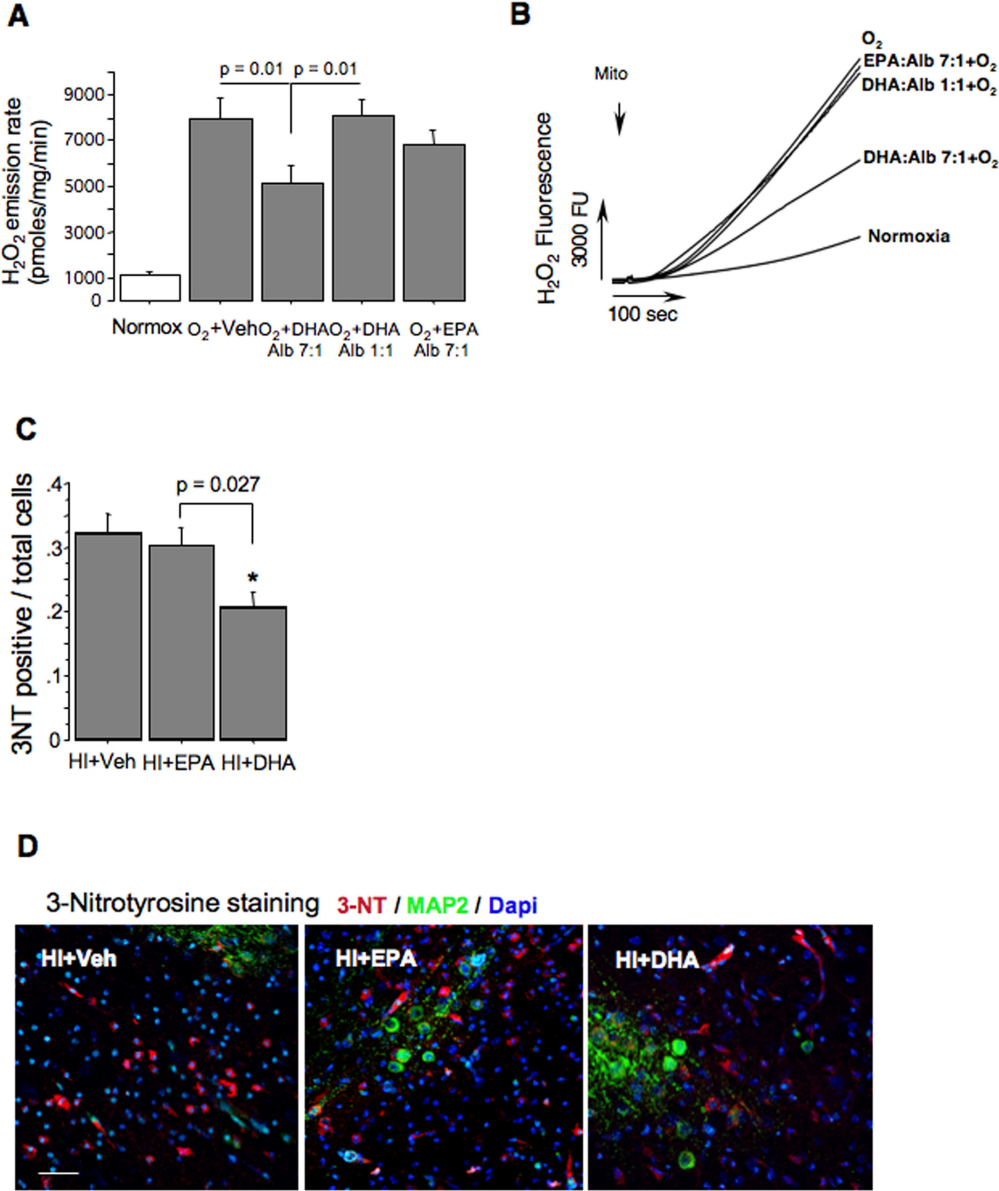
Mitochondrial production of ROS in vitro and markers for oxidative brain injury in vivo. (A) Mitochondrial H_2_O_2_ emission rates in Normox; O_2_+Veh; O_2_ +DHA:Alb 7:1; O_2_ +DHA:Alb 1:1; and O_2_ +EPA:Alb 7:1. N = 5 in all groups (B) Representative tracing of mitochondrial H_2_O_2_ production (groups are indicated). (C) 3-nitrotyrosine immunopositive cells / total cells ratio in HI+Veh, HI+EPA and HI+DHA. N = 4 in all groups. * p = 0.02. (D) Representative images of 3-nitrotyrosine staining in HI+Veh, HI+EPA and HI+DHA mice.

As well, hyperoxia dramatically increased mitochondrial H_2_O_2_ emission rates (Fig 5A and 5B). Following incubation with DHA:albumin 7:1, hyperoxic mitochondria significantly (p = 0.01) reduced their H_2_O_2_ emission rate compared to the same mitochondria exposed to O_2_ or O_2_+DHA:albumin 1:1 (Fig 5A and 5B). No effect on mitochondrial H_2_O_2_ generation rate was detected in organelles incubated with EPA:albumin 7:1 (Fig 5A and 5B), S2 Data.

### Tri-DHA but not tri-EPA attenuates oxidative brain injury following HI insult

To translate our in vitro data into the in vivo model, we examined cerebral tissue for evidence of oxidative damage in HI-mice. At 24h of reperfusion, HI+tri-DHA mice exhibited significantly fewer cells immunopositive for 3-NT, a marker of protein oxidation, compared to the HI+tri-EPA or HI+Veh groups (Fig 5C and 5D).

### HI-mice treated with tri-DHA exhibited significant accumulation of DHA derived bioactive metabolites in brain

**Fig 6:**
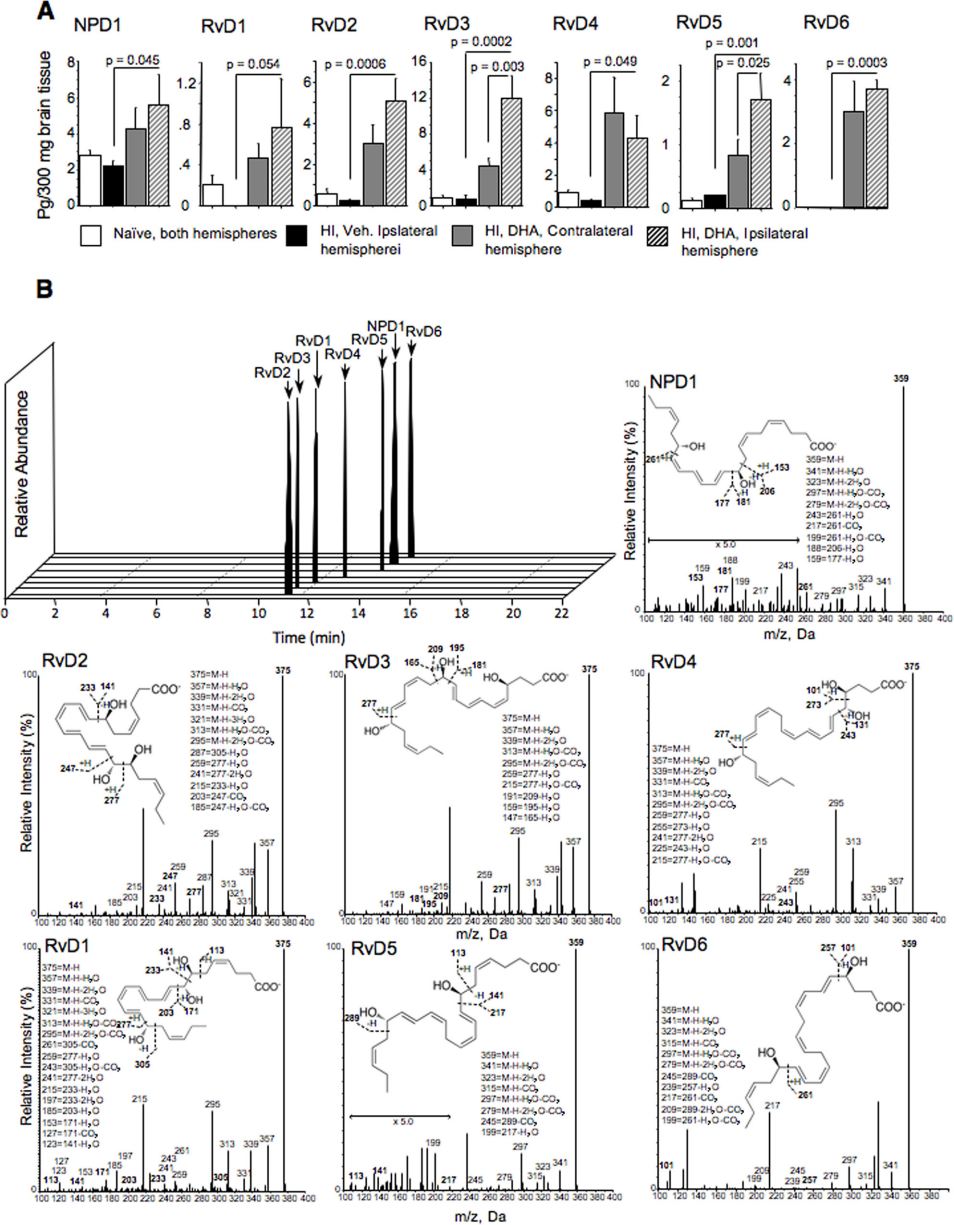
DHA-metabolites content in the brain. (A) Cerebral content of DHA metabolites in naïve (n = 4) or HI-mice treated with either vehicle or tri-DHA (n = 3). (B) DHA metabolites spectra used for calculation of their concentration in the brains of mice (groups are indicated above).

At two hours of reperfusion (one hour following a completion of tri-DHA treatment), we found significantly greater accumulation of DHA derived bioactive metabolites, NPD1, and all RvD series in the ipsilateral hemisphere compared to that in the vehicle-treated littermates (Fig 6A), S3 Data. Fig 6B shows the NPD1 and all six RvD spectra obtained from brains of vehicle and tri-DHA treated HI-mice which were used to determine their concentrations. There was a tendency for increase in these DHA derivatives in the contralateral unaffected hemisphere suggesting that increases in these compounds might in part relate to a “systemic” release from exogenous DHA. Of note, in the HI-mice treated with tri-DHA, accumulation of some DHA derived bioactive metabolites (RvD3, RvD5) was significantly greater in the ipsilateral, ischemic hemisphere compared to that in the contralateral, unaffected hemisphere (Fig 6A).

## Discussion

Our study provides original *in vivo* and *in vitro* evidence for (a) permanent neurological benefits and (b) mitochondria-associated mechanisms of neuroprotection afforded by post-treatment with tri-DHA after HI brain injury in neonatal mice. We show that, during evolution of reperfusion injury, administration of tri-DHA was coupled with increased DHA content in brain mitochondria, the event associated with preservation of mitochondrial tolerance to Ca^2+^-induced permeabilization. We also demonstrate significant accumulation of DHA derived bioactive metabolites in the HI-affected brains in mice treated with tri-DHA. This suggests that exogenous DHA and its metabolites are transferred to, and accumulate in HI-affected brain tissue and mitochondria, modifying mitochondrial membrane response to Ca^2+^ overload. Our in vitro experiments demonstrate that direct interaction of DHA with mitochondria diminished hyperoxia-induced surge in mitochondrial ROS production and preserved mitochondrial Ca^2+^ buffering capacity.

We have reported that tri-DHA significantly reduced brain injury assessed at 24h and 8 weeks after HI [12]. However, it was not determined whether this reduction in the extent of injury translates into long-term neurofunctional benefits. We extended our observational strategy by assessment of long-term neurofunctional outcomes in HI-mice treated with tri-DHA or tri-EPA. Our study showed that only DHA but not EPA exerted permanent long term neuroprotection, as evidenced by the reduced anatomical extent of injury and by better preservation of navigational memory in adulthood. In models of stroke and myocardial infarction in mature rodents, DHA also conferred better organ protection than EPA [19,20]. Pan et al. found that pre-treatment with DHA for 6 weeks prior to HI insult significantly attenuated brain injury [20].In the neonatal rat model of HI brain injury, pre-treatment with albumin-complexed DHA improved neurofunctional recovery, although no neuroanatomically-identifiable benefit was detected [21]. In the same model, DHA:albumin complexes markedly augmented the neuroprotective effects of hypothermia [22]. Thus, our data are in agreement with other reports on neuroprotective actions of DHA in the setting of perinatal HI-brain injury.

The mammalian central nervous system contains relatively high levels of DHA but very low levels of EPA [23]. This suggests that in the brain, EPA might be a precursor of DHA synthesis.^3^ Daily EPA IP injection for one week conferred neuroprotection in a model of transient bilateral carotid artery occlusion [24]. Chronic EPA administration for 4 weeks improved memory function and decreased neuronal loss in the same rodent model [24]. Perhaps delayed tissue distribution of EPA associated with chronic administration is required to achieve beneficial effects against cerebral ischemia. However, in the setting of acute treatment, tri-EPA does not affect the propagation of HI brain injury.

Alterations of FA levels in plasma membranes change fluidity, altering the function of membrane-associated proteins [19,25]. Dietary supplementation of n-3 FAs increased mitochondrial content of DHA and EPA in cardiac tissues [19]. Importantly, only changes in mitochondrial DHA but not EPA were associated with significantly greater Ca^2+^ buffering capacity in cardiac mitochondria [19]. In our study, post-HI administration of DHA also significantly increased mitochondrial DHA content assessed at the time when secondary energy failure takes place in this model.^6^ Only DHA-enriched brain mitochondria exhibited improved tolerance to Ca^2+^-induced membrane permeabilization. Post-ischemic Ca^2+^-induced permeabilization of mitochondrial membranes is currently viewed as the mechanism of secondary energy failure [6,26,27]. Our data suggest that post-HI enrichment of cerebral mitochondria with DHA may account for the attenuation of the severity of secondary energy failure, as DHA-enriched organelles were more resistant to Ca^2+^-induced mitochondrial permeabilization. Our in vitro data supports this point, since incubation of cerebral mitochondria with DHA-saturating albumin complexes significantly increased DHA content in these organelles. This effect was translated into significantly greater preservation of mitochondrial Ca^2+^ buffering capacity which was markedly altered by hyperoxic stress. Another potential neuroprotective mechanism of post-HI tri-DHA administration may be exerted by DHA derived bioactive metabolites, such as NPD1 and/or the RvD family. This hypothesis is supported by our results, demonstrating a significant accumulation of these metabolites in the HI-affected brains following treatment with tri-DHA. Our data provide an important rationale for future studies in testing potential neuroprotective actions and mechanisms related to a number of DHA-derivatives. While neuroprotective properties of NPD1 in murine models of focal stroke has been reported [8], our data on the robust elevations of RvD compounds in the ipsilateral hemisphere now highlight potential neuroprotective effects of these DHA metabolites. It has been shown, that RvD2, RvD3 and RvD4 exert strong anti-inflammatory actions [28,29], protecting murine lungs against ischemia-reperfusion injury [30].

Oxidative stress plays a key mechanistic role in reperfusion injury and, in particular, induction of secondary brain injury [2,6,31,32] By mimicking hyperoxic re-oxygenation/reperfusion, our *in vitro* paradigm demonstrated that cerebral mitochondria dramatically accelerate generation of ROS known to cause oxidative injury to the post-ischemic brain and other tissues [2,6,33]. The same experiment showed that enrichment of mitochondria with DHA significantly limited hyperoxia-induced increases in mitochondrial ROS production. These in vitro data suggest a potential mechanism underlying the neuroprotective effects of acute tri-DHA post-treatment in vivo—exogenous DHA incorporates into post-HI mitochondria, and this limits a reperfusion-driven acceleration in mitochondrial ROS release, and attenuates the oxidative injury which sensitizes mitochondria to Ca^2+^-mPTP. This potential sequence of events is supported by significant attenuation of oxidative brain damage in HI+tri-DHA mice compared to their HI+tri-EPA treated littermates.

A limitation of our study is the absence of direct evidence for the exogenous origin of mitochondrial DHA-enrichment following post-treatment with tri-DHA. Therefore, our data can only suggest that mitochondrial enrichment with DHA in vivo was due to administration of exogenous tri-DHA. However, our in vitro experiments clearly demonstrate that upon interaction of mitochondria, the DHA incorporates into organelles and this improves mitochondrial abilities to buffer excess of Ca^2+^. The specificity of DHA-mitochondria interaction in this effect is supported by unchanged mitochondrial phenotypes when the same dose of DHA was used but at DHA:albumin molar ratio of 1:1. Finally, significant elevation of DHA derived bioactive metabolites t in the brains of only DHA-treated HI-mice strongly supports exogenous origin of the DHA and its derivatives in the affected tissue.

Another limitation is that we did not explore all potential therapeutic mechanisms of DHA. It is possible that other reported mechanisms of post-HI neurodegeneration, glutamate excitotoxicity, neuroinflammation and BAX induced apoptosis are affected by DHA and/or biologically active DHA metabolites. We believe that these limitations do not reduce the significance of our work which is the first to demonstrate the permanent nature of DHA-driven neuroprotection with an emphasis on post-ischemic mitochondria as the main therapeutic target. Thus, acute post-treatment with tri-DHA, and DHA derived bioactive metabolites (NPD1, RvDs) should be considered for future research on the translation of this very promising neuroprotective strategy into clinical practice.

## Supporting Information

S1 Data Raw data on water maze performance in adult mice subjected to HI-insult and treated with DHA, EPA or vehicle. (XLS)

S2 Data Raw data. Mitochondrial ROS generation rate in vitro.
(XLSX)

S3 Data Raw data on DHA metabolites content in the brain following HI-insult and treatment with DHA (XLSX)
